# The Hunterian Oration, Delivered at the Royal College of Surgeons in London on the 14th February, 1843

**Published:** 1843-04

**Authors:** 


ART. II.—*The Hunterian Oration delivered at the Royal College of Surgeons in London, on the* 14*th of February*, 1843. By J. M. ARNOTT, Surgeon to the Middlesex Hospital.—*London*, 1843. 8vo, pp. 37.

THIS is an excellent oration. Besides containing some interesting observations on the prescribed and inexhaustible theme of these annual discourses, JOHN HUNTER,—it gives the best account yet published of the life and labours of Sir Charles Bell, and also some interesting particulars of Baron Larrey. The materials are clearly digested,—the style is good, and the whole production is creditable to Mr. Arnott as a surgeon and a scholar.

